# Atypical Mitotic Figures Are Prognostically Meaningful for Canine Cutaneous Mast Cell Tumors

**DOI:** 10.3390/vetsci11010005

**Published:** 2023-12-20

**Authors:** Christof A. Bertram, Alexander Bartel, Taryn A. Donovan, Matti Kiupel

**Affiliations:** 1 Institute of Veterinary Pathology, University of Veterinary Medicine Vienna, 1210 Vienna, Austria; 2 Institute for Veterinary Epidemiology and Biostatistics, Freie Universität Berlin, 14163 Berlin, Germany; alexander.bartel@fu-berlin.de; 3 Department of Anatomic Pathology, The Schwarzman Animal Medical Center, New York, NY 10065, USA; taryn.donovan@amcny.org; 4 Veterinary Diagnostic Laboratory, Michigan State University, Lansing, MI 48910, USA

**Keywords:** atypical mitotic figure, cell division, digital microscopy, dog, mitotic count, mast cell tumor, mitotic index, outcome, prognosis, survival time

## Abstract

**Simple Summary:**

The aim of this study was to evaluate a new microscopic parameter (atypical mitotic figures) in canine cutaneous mast cell tumors regarding the ability to predict patient survival (prognosis). Mast cell tumors are one of the most common skin tumors in dogs. Counting the number of tumor cells undergoing division (mitotic figures) is of prognostic importance for this tumor type. While normal cells have a highly regulated cell division (normal mitotic figures), tumor cells might exhibit errors during chromosome separation into daughter cells. These errors can be identified microscopically as atypical mitotic figures and are described as a malignancy criterion for some human tumors. In this study, we have shown that a high number of atypical mitotic figures in canine cutaneous mast cell tumors is predictive of shorter patient survival. As compared to enumerating all mitotic figures, increased numbers of atypical mitotic figures (≥3 per 2.37 mm^2^) had a higher specificity for tumor-related death. These findings should be validated in future studies.

**Abstract:**

Cell division through mitosis (microscopically visible as mitotic figures, MFs) is a highly regulated process. However, neoplastic cells may exhibit errors in chromosome segregation (microscopically visible as atypical mitotic figures, AMFs) resulting in aberrant chromosome structures. AMFs have been shown to be of prognostic relevance for some neoplasms in humans but not in animals. In this study, the prognostic relevance of AMFs was evaluated for canine cutaneous mast cell tumors (ccMCT). Histological examination was conducted by one pathologist in whole slide images of 96 cases of ccMCT with a known survival time. Tumor-related death occurred in 11/18 high-grade and 2/78 low-grade cases (2011 two-tier system). The area under the curve (AUC) was 0.859 for the AMF count and 0.880 for the AMF to MF ratio with regard to tumor-related mortality. In comparison, the AUC for the mitotic count was 0.885. Based on our data, a prognostically meaningful threshold of ≥3 per 2.37 mm^2^ for the AMF count (sensitivity: 76.9%, specificity: 98.8%) and >7.5% for the AMF:MF ratio (sensitivity: 76.9%, specificity: 100%) is suggested. While the mitotic count of ≥ 6 resulted in six false positive cases, these could be eliminated when combined with the AMF to MF ratio. In conclusion, the results of this study suggests that AMF enumeration is a prognostically valuable test, particularly due to its high specificity with regard to tumor-related mortality. Additional validation and reproducibility studies are needed to further evaluate AMFs as a prognostic criterion for ccMCT and other tumor types.

## 1. Introduction

Neoplastic cell proliferation is one of the hallmarks of tumorigenesis. In diagnostic veterinary pathology, the number of dividing cells (mitotic figures, MFs) per tumor area, referred to as the mitotic count (MC), is commonly determined in routine histological sections. For some canine and feline neoplastic entities, including canine cutaneous mast cell tumors (ccMCT), the MC has been shown to be prognostically meaningful [1,2,3,4,5,6,7,8,9]. For ccMCT, the MC is also part of the well-established, two-tier histologic grading system published in 2011 [10] and the older, three-tier histologic grading system published in 1984 [11], and thus is routinely determined for each diagnostic case.

While cell division is usually a highly regulated process in non-neoplastic cells (histologically characterized by normal MF morphology), errors in chromosome segregation during the mitosis phase of the cell cycle may occur in neoplastic cells, resulting in aberrant chromosome constitutions (aneuploidy) of the daughter cells [12]. These errors in cell division may be seen histologically as atypical MFs (AMFs) [13,14]. The biological relevance of AMFs is the accumulation of many genetic alterations through aneuploidy, which can promote tumor progression [12].

Normal MF morphologies depend on the stage of the M phase of the cell cycle, which is separated into prometaphase, metaphase (including ring phase morphology), anaphase and telophase [13,14]. AMFs are any MF not consistent with a normal division, and include polar asymmetry (bipolar size asymmetry and spindle multipolarity) and segregation abnormalities (chromosome or fragment lagging and chromosome bridging) as well as more complex abnormalities [12].

Few publications on neoplasms in humans have shown the prognostic value of AMFs for breast cancer [15,16], gastrointestinal stromal tumors [17], mesothelioma [18], pancreatic cancer [19], uterine smooth muscle tumors of uncertain malignant potential [20], and thyroid cancer [21]. These studies have correlated the presence or number of AMFs and/or the AMF to MF ratio with poor patient outcome. There is currently no study for neoplasms in animals on this topic. We hypothesized that AMFs have prognostic value for ccMCT and that combining AMFs with the MC has an added value for prognostication.

## 2. Materials and Methods

### 2.1. Study Population and Histological Sections

For this study, histological sections of 96 cases of ccMCT were used. Tumors diagnosed as ccMCT (located within the dermis with or without extension into deeper layers) at the Veterinary Diagnostic Laboratory (VDL) of Michigan State University (MSU) were identified, and questionnaires were sent to the submitters of the tissue samples in order to obtain outcome information of the patients (including survival time and suspected cause of death). Cases were excluded if: (1) a complete questionnaire was not returned, (2) the patients were lost to follow-up earlier than 12 months after surgical removal (with the exception of death occurring within 12 months), (3) the patients received any further treatment beyond the primary surgical removal of the tumor, or (4) sufficient tumor tissue for histological re-evaluation was not available. The cause of death was based on the clinician’s interpretation of the case, and confirmation of the cause of death with pathological examination was not available.

Histological sections were routinely processed at the VDL and were stained with Hematoxylin and Eosin. One representative section per case was selected and digitized with the Pannoramic Scan II whole slide image scanner (3D Histech) at a magnification of 400× (image resolution: 0.25 µm per pixel).

### 2.2. Histological Evaluation

**Figure 1:**
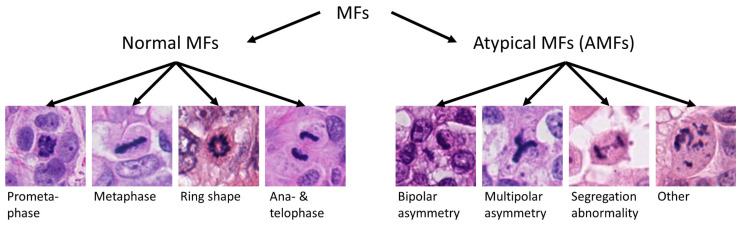
Examples of the different mitotic figure (MF) morphologies from normal and atypical cell division. The image patches are from publicly available datasets on human breast cancer [26,27].

Using the software SlideRunner [22], a mitotic hotspot tumor location was selected in the whole slide image by a pathologist (C.A.B.) and marked by a rectangular (4:3 ratio) annotation with a size of 2.37 mm^2^ (equivalent to 10 high power fields, HPF), as previously described [23]. For the region of interest, areas with necrosis and underlying ulceration were avoided according to current recommendations [13,24]. Within these selected tumor areas, a pathologist (C.A.B.) independently identified all MFs (bounding box annotation) and subsequently manually labeled them as normal MFs, AMFs (all morphological groups), and AMF with multipolar asymmetry, separately, according to predefined classification criteria (Figure 1) [13]. The pathologist was blinded to the survival information and had some experience in classifying normal against atypical MFs through a previous study [25]. For each case, the MC (number of all MFs per 2.37 mm^2^ including normal MFs and AMFs, according to current recommendations [13,24]), the AMF count (number of AMFs per 2.37 mm^2^), the AMF to MF ratio ($\frac{AMF\;count-1}{Mitotic\;count}$), and the multipolar AMF count (number of AMFs with multipolar asymmetry per 2.37 mm^2^) were determined. For the ratio, the AMF count (if >0) was subtracted by one in order to reduce the impact of a single AMF in cases with low mitotic activity. Cases without a single MF were set to have a ratio of 0% (division by zero is not possible).

The two-tier histological grade was determined by one pathologist (C.A.B.) according to the described methods [10]. The grade is considered high if one of the following parameters is above the threshold: MC (threshold: ≥7), number of multinucleated cells in 10 HPF (threshold: ≥3), number of bizarre nuclei in 10 HPF (threshold: ≥3), and karyomegaly (present). For this study, the MC, as determined above, was considered first, and, if below the threshold, further parameters were evaluated in the whole slide image.

### 2.3. Statistical Analysis

Statistical analysis was performed with R version 4.2.2 (R Foundation, Vienna, Austria). As the primary outcome metric, tumor-related mortality was defined as the event of death considered by the clinician to be associated with the ccMCT and occurring at any time within the patient’s follow-up period. Tumor-specific survival time (in months) is the time from tumor surgery with submission of the tumor sample for histological examination until tumor-related death (cases that died due to other causes or were lost to follow-up after 12 months were censored at that respective time). As a secondary outcome metric, we also used all-cause mortality within the first 12 months of the follow-up period, which is the minimum follow-up for each patient (see exclusion criteria), and overall survival time (time until death of any cause in months).

The MC is considered an appropriate benchmark for the prognostic tests on AMFs (AMF count, AMF to MF ratio, presence of multipolar AMFs) as all are quantitative (and thus the same statistical analysis can be conducted), and all are solitary morphological criteria and can be conducted in routine hematoxylin and eosin stained sections. In contrast, the histological grading systems for ccMCT [10,11] are categorial and comprise numerous morphological parameters (including the MC) and are thus theoretically superior to a solitary test.

Receiver operating characteristic (ROC) curves (sensitivity and specificity plotted for numerous thresholds) were drawn in order to calculate the area under the ROC curve (AUC) for tumor-related and overall mortality. The AUC is the recommended statistical test to measure the overall discriminative ability of a numerical prognostic test independent of a specific threshold with a value of AUC = 1 indicating perfect discriminative ability and a value of AUC = 0.5 indicating a lack of discriminative ability [28]. Scatterplots (tumor-related mortality) were used to determine binary prognostic cut-offs for the AMF count, AMF to MF ratio, and multipolar AMF count. The binary classification for an MC of 0–5 (good prognosis) vs. ≥6 (poor prognosis) was used according to the threshold proposed by Romansik et al. [2]. Based on this classification, Kaplan–Meier curves and hazard ratios (univariate cox proportional models) with censoring of cases that were lost to follow-up and, in the case of tumor-specific survival time, died due to a tumor-unrelated cause (tumor-specific survival time), were determined. Additionally, sensitivity, specificity, and the positive predictive value were calculated.

## 3. Results

### 3.1. Study Population

In the 96 cases, there were 51 spayed females, 33 castrated males, 7 intact females, and 5 intact males. The dogs were younger than 2 years (N = 3), between 3–5 years (N = 27), between 6–9 years (N = 40), or older than 10 years (N = 26). Breeds of the dogs included: mixed breed (N = 21), Labrador retriever (N = 21), boxer (N = 11), golden retriever (N = 10), pug (N = 4), basset hound (N = 4), Boston terrier (N = 3), cocker spaniel (N = 3), and other breeds (N = 19).

Death within the follow-up period occurred in 24/96 cases, of which 13 were considered tumor-related and 11 were attributed to tumor-unrelated causes. Based on the 2011 two-tier grading system, 78 cases were determined as low grade (81%) and 18 cases as high grade (19%). Of the 18 high-grade cases, 15 were assigned based on the MC (≥7) and three cases with a low MC (0–6) had karyomegaly (N = 2) or multinucleation (N = 1).

**Figure 2:**
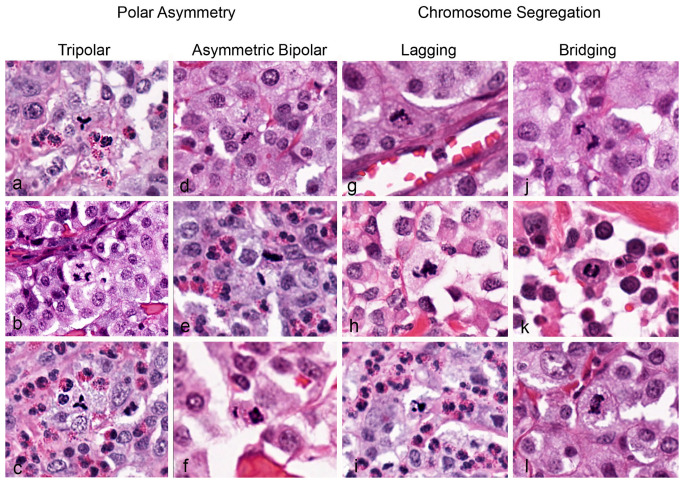
Atypical mitotic figures, canine cutaneous mast cell tumor. Each column contains examples of four types of AMF. (**a**–**c**) Are examples of tripolar morphology, with formation of three spindle poles and a characteristic “Y” shape at metaphase. In anaphase (**b**), the chromosome clusters are pulled in three separate directions, which may culminate in the formation of three daughter cells. (**d**–**f**) Are examples of bipolar asymmetry, in which the two chromosome clusters are of unequal size, typically observed in anaphase. (**g**–**i**) Are examples of chromosome lagging, in which chromosomes are not in contact with the larger central chromosome aggregate. (**j**–**l**) Are examples of chromosome bridging, in which chromosomes stretch from one anaphase pole to the other (touching both) during anaphase or telophase. All images were taken from whole slide images with a resolution of 0.25 µm per pixel.

**Figure 3:**
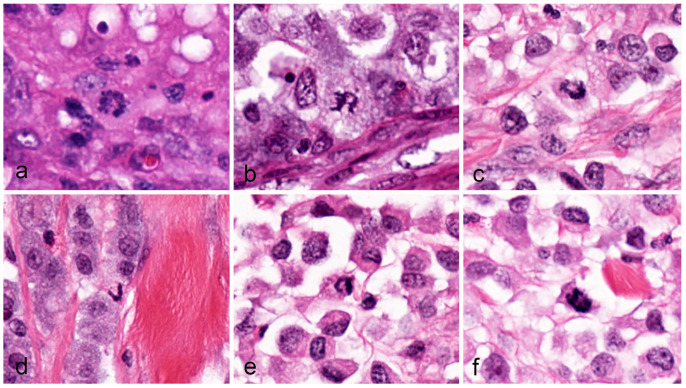
Presumptive atypical mitotic figures, canine cutaneous mast cell tumor. Images (**a**–**f**) are structures of unknown categorization and are of uncertain significance. These structures appear as “C”, “U”, or “V” shapes and may represent a sectioning artifact of a normal or atypical mitotic figure, a segregation abnormality, or a combination of abnormalities during the mitosis phase. All images were taken from whole slide images with a resolution of 0.25 µm per pixel.

**Figure 4:**
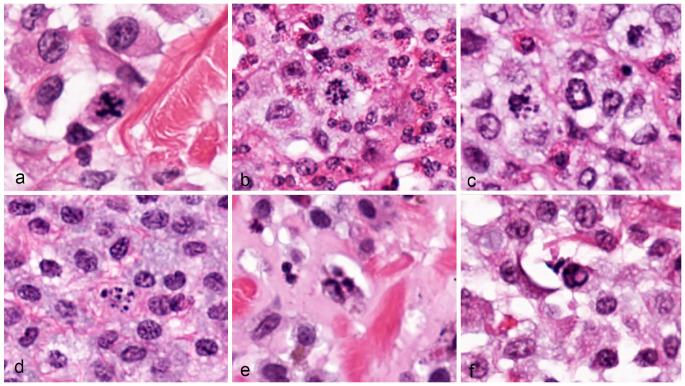
Atypical mitotic figures, canine cutaneous mast cell tumor. Images (**a**–**f**) are structures with multiple possible interpretations or are of unknown categorization. (**a**) This structure may represent a multipolar AMF or a central chromosome bridge with two spindle poles. (**b**,**c**) Are “dispersed” AMF, in which chromosome material appears to be scattered in the cytoplasm, with no recognizable mitotic structure. (**d**) May also represent a dispersed AMF; however, this would be impossible for a pathologist to differentiate from a mitotic-like figure (karyorrhexis) with HE staining. (**e**,**f**) Are of unknown categorization. All images were taken from whole slide images with a resolution of 0.25 µm per pixel.

In all tumor cases combined (2.37 mm^2^ tumor area per case), 650 MFs were identified, of which 103 (15.8%) were classified as AMFs. The AMF count ranged between 0 and 15 and the AMF to MF ratio ranged between 0 to 37.5% (Supplemental Figure S1). Multipolar AMFs were found only in eight cases (count range: 0–4). In the authors’ experience, some MF morphologies are difficult to classify into the different normal phases and AMF categories as summarized in Figure 1. Examples of clear and doubtful AMFs (by consensus of C.A.B. and T.A.D.) from the study cases are depicted in Figure 2, Figure 3 and Figure 4.

### 3.2. Prognostic Value of AMFs

The AMF count (AUC = 0.859) and the AMF to MF ratio (AUC = 0.880), were strongly discriminative for tumor-related death, similar to the MC (AUC = 0.885; Table 1). Results for all-cause mortality are listed in Table 2.

**Figure 5:**
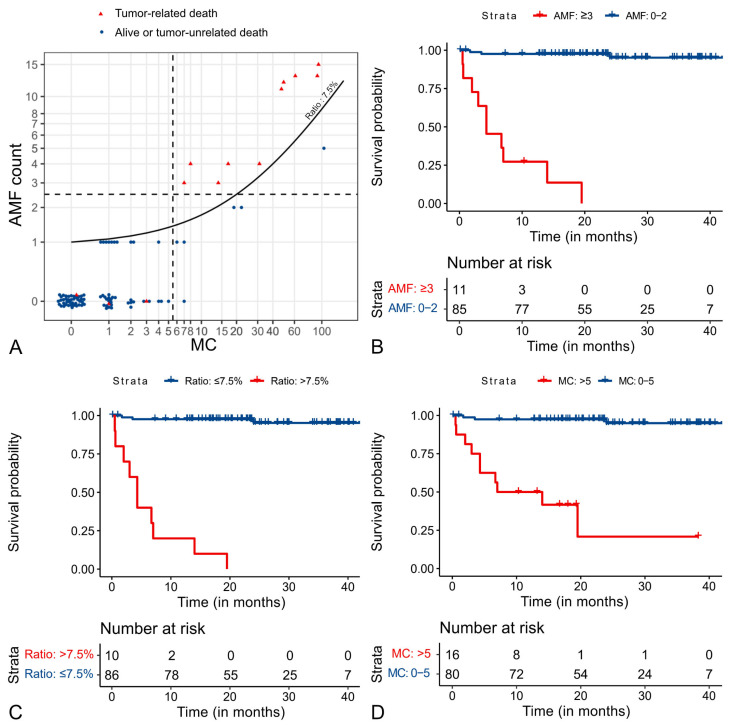
Survival analysis (all-cause mortality/tumor-specific survival time) of 96 canine cutaneous mast cell tumor cases. (**A**) Scatter plots with suggested/published thresholds (broken lines) for the atypical mitotic figure (AMF) count (*y*-axis) and routine mitotic count (*x*-axis). The red triangles represent cases with tumor-related death and blue dots represent cases that did not die due to the mast cell tumor. The line in the graph (appears curved due to the log transformation of the axis) is the threshold for the AMF to mitotic figure ratio of 7.5%. (**B**) Kaplan–Meier curve for the AMF count. (**C**) Kaplan–Meier curve for the AMF to mitotic figure ratio. (**D**) Kaplan–Meier curve for the routine mitotic count (MC).

Based on our scatterplot analysis (Figure S1), we propose prognostic thresholds of an AMF count of ≥3 and an AMF to normal MF ratio of >7.5%. These thresholds resulted in a sensitivity and specificity of the AMF count of 76.9% and 98.8%, respectively, and of the AMF to MF ratio of 76.9% and 100%, respectively, and 76.9% and 92.7% for the MC. Interestingly, six cases with a high MC of ≥6 (37.5%) did not die of a tumor-related cause (false positives), of which five (83%) had a low AMF count of <2, and six (100%) had a low AMF to MF ratio of <7.5% (true negatives; Figure 5A). This results in a higher positive predictive value of the AMF count (90.9%) and the AMF to MF ratio (100%) as compared to the MC (62.5%). Cases with a low MC (0–5) never had a high AMF count or AMF to MF ratio.

Kaplan–Meier curves show that dogs with high values (above the threshold of the respective prognostic test) have a median tumor-specific survival time of less than 10 months, while the median survival time of the low-value group (below the threshold) was not reached for any of the three tests (Figure 5B–D). Hazard ratios show that the AMFs are strong predictors of survival time (Table 1 and Table S1).

The presence of any multipolar AMFs had a good overall prognostic value in this study with a hazard ratio of 20.6 (95% CI: 6.7–62.6). While there was only one case with a false positive classification based on the presence of a multipolar AMF (positive predictive value: 87.5%, specificity: 98.8%), only 7/13 cases (sensitivity: 53.8%) that died due to a tumor presented with at least one multipolar AMF in the enumerated tumor area.

Results of the prognostic value of the 2011 two-tier grading system is provided in Supplemental Tables S1 and S2.

## 4. Discussion

AMF enumeration is a practical quantitative test that can be conducted in routine histological sections simultaneously with the MC and is thus of potential interest for routine histologic tumor prognostication. This study demonstrates the high prognostic value of the AMF count, the AMF to MF ratio, and the presence of multipolar AMFs for ccMCT, similar to previous studies on cancer in humans [15,16,18,19,20,21]. The value of AMF quantification at the proposed thresholds seems to be a high specificity and positive predictive value as compared to the MC due to a markedly lower number of false positives (cases with high values but without tumor-related death). Our data suggest that the AMF count and AMF to MF ratio may be used to detect false positive cases based on a high MC; however, validation of these results in an independent study population is needed. Similarly, the presence of multipolar AMFs had a high positive predictive value for tumor-related mortality (i.e., high likelihood of death due to the ccMCT if present) in our study population and might be used to support the suspicion of an aggressive tumor. A larger study population is required to show the potential benefits of AMF enumeration as compared to the routine MC and other prognostic tests.

While the frequency of mitosis (measured by the MC) and the occurrence of errors in the highly regulated chromosome segregation process (AMF count) are two separate mechanisms of tumorigenesis [29,30], it seems reasonable that a combination of the two might be biologically more meaningful than each parameter alone. Increased mitosis occurs mostly due to dysregulation of the G_1_/S checkpoint at the beginning of the cell cycle [29], and the chromosome segregation errors are associated with dysregulation of the mitotic checkpoint (also known as the spindle assembly checkpoint) during the M phase at the end of the cell cycle [30].

This is one of the first studies in veterinary medicine that has evaluated the prognostic value of the MC in whole slide images [31]. The use of digital microscopy for enumeration of mitotic figures and other cellular features has been discussed controversially [13,31]. Our data support that the threshold proposed by Romansik et al. [2] can be applied to digital microscopy for ccMCT. The impact on the ability to identify AMFs with digital image evaluation (e.g., lack of fine focus) is currently unknown. Validation of digital microscopy for this task is needed.

The limitation of the AMF to MF ratio is that a single AMF in a tumor with a low MC dramatically influences the ratio. It seemed reasonable to eliminate the cases with a single AMF and absent or one normal MF (occurring in 9/96 cases), which would result in a ratio of 50% or 100%, as these had a good prognosis in our study. This finding guided us to reduce the AMF count by one for the calculation of the ratio. An alternative might be to evaluate a minimum number of MFs per case by enumerating larger tumor areas (>2.37 mm^2^) in tumors with low mitotic activity, which could become feasible with the use of deep learning-based algorithms [25]. In the present study, the tumor area evaluated was selected based on mitotic activity. It is currently unknown whether there is a spatial association between mitotic activity and the occurrence of AMFs. The distribution of AMFs throughout the tumor sections likely has an impact on enumeration. Another interesting aspect for future studies is to evaluate the prognostic value of specific AMF types. In one study on human pancreatic tumors, the presence of a multipolar AMF had a higher prognostic value than the presence of any AMF [19]. The potential benefit of multipolar AMFs is that they are usually easily detectable and have characteristic morphology [13] (thus, they may have less inter-pathologist variation in identification as compared to some other AMF types). Spindle multipolarity is associated with the presence of supernumerary centrosomes, with highly uncoordinated sister chromatid separation, representing a particularly severe form of chromosome segregation error [12]. While the presence of multipolar AMFs had a prognostic value in this study, several cases with a poor outcome did not have multipolar AMFs, suggesting that this test might be more specific than sensitive. Future studies need to evaluate whether weighing specific types of AMFs or the elimination of less relevant or less easily detectable AMFs is reasonable, based upon their biological importance and impact on prognostication. In particular, it is unclear how often bipolar asymmetry may actually reflect uneven cut angles through a normal anaphase/telophase MF. Similarly, we have identified some MFs with a V-shape or U-shape, for which the underlying mechanism is unknown to us, and, while annotated as “other” AMFs in the present study, we are concerned whether they could be normal MFs with an unusual two-dimensional presentation. For a U-shape, an uneven cut angle of a ring shape (normal) MF could be possible. Guidelines for the interpretation of these structures are needed. For example, bipolar asymmetry can be more easily interpreted by comparing the two chromosome clusters and establishing a percentage difference between the two clusters (e.g., one cluster must be at least 50% larger than the other in order to count as bipolar asymmetry). The histological morphology of MFs is known to be quite variable and has overlap with non-mitotic imposters (such as cells with pyknotic and hyperchromatic nuclei) [13] leading to inter- and intra-observer variability of the MC [23]. Beyond that, we have experienced some difficulty in classifying the MFs into different morphologies (normal mitosis phases and atypical classes). In the current literature, there are few studies that have evaluated the inter- and intra-observer reproducibility of classifying normal MFs and AMFs more systematically [25,32]. Based on our experience, chromosome lagging or chromosome fragments seem to be particularly difficult to distinguish from normal mitotic “spikes” or other intracytoplasmic basophilic material. Dispersed chromatin throughout the cytoplasm may be difficult to distinguish from apoptotic cells (karyorrhexis); however, based on the authors’ experience from previous studies [23], they often immunohistochemically react with pHH3-antibodies that are specific to the M phase of the cell cycle. Small chromosome dots in the center of cytoplasm likely represent perpendicular sections through linear chromosome aggregates (normal throughout metaphase to telophase) and should not be counted as AMFs. Further studies that evaluate the inter- and intra-rater reproducibility of AMF enumeration are needed.

## 5. Conclusions

In conclusion, AMFs were shown to have a high prognostic value in this series of ccMCT. There might be an added value of combining the routine MC with the AMF count or AMF to MF ratio due to the high specificity and positive predictive value for tumor-related mortality. Future studies are needed for validation of the prognostic value for ccMCT, identification of AMF types with particular biological relevance, investigation of AMFs in additional animal tumors, and exploration of the inter- and intra-observer reproducibility between several pathologists.

## Figures and Tables

**Table 1:** Results of statistical analysis regarding the prognostic value (tumor-related mortality/tumor-specific survival time) of the atypical mitotic figure (AMF) count, the AMF to mitotic figure (MF) ratio, and the mitotic count for 96 canine cutaneous mast cell tumors.

Statistical Analysis	AMF Count	AMF to MF Ratio	Mitotic Count
Area under the ROC curve (95% CI)	0.859 (0.719–0.999)	0.880 (0.757–1.0)	0.885 (0.763–1.0)
Prognostic classification ranges	0–2 vs. ≥3	0–7.5% vs. >7.5%	0–5 vs. ≥6
Sensitivity (95% CI) *	76.9% (46.2–94.9%)	76.9% (46.2–94.9%)	76.9% (46.2–94.9%)
Specificity (95% CI) *	98.8% (93.5–99.9%)	100% (95.6–100%)	92.7% (84.9–97.3%)
Positive predictive value (95% CI) *	90.9% (58.7–99.8%)	100% (69.2–100%)	62.5% (35.4–94.8%)
Hazard ratio (95% CI) *	79.6 (16.6–382.7)	90.7 (19.0–432.0)	30.6 (7.9–118.6)

95%CI, 95% confidence interval; * statistical analysis with case classification based on the applied threshold (see third line of this table).

**Table 2:** Results of statistical analysis regarding the prognostic value (all-cause mortality at 12 months after surgery/overall survival time) of the atypical mitotic figure (AMF) count, the AMF to mitotic figure (MF) ratio, and the mitotic count for the 96 canine cutaneous mast cell tumors.

Statistical Analysis	AMF Count	AMF to MF Ratio	Mitotic Count
Area under the ROC curve (95% CI)	0.688 (0.519–0.858)	0.727 (0.565–0.890)	0.744 (0.596–0.891)
Prognostic classification ranges	0–2 vs. ≥3	0–7.5% vs. >7.5%	0–5 vs. ≥6
Sensitivity (95% CI) *	50.0%	50.0%	50.0%
Specificity (95% CI) *	96.3%	97.5%	90.0%
Positive predictive value (95% CI) *	72.7%	80.0%	50.0%
Hazard ratio (95% CI) *	18.3 (7.1–47.2)	22.3 (8.9–57.7)	7.1 (3.0–16.8)

95%CI, 95% confidence interval; * statistical analysis with case classification based on the applied threshold (see third line of this table).

## Data Availability

The data presented in this study are available on request from the corresponding author.

## Supplementary Materials

The following supporting information can be downloaded at: https://www.mdpi.com/article/10.3390/vetsci11010005/s1, Figure S1: Scatterplot analysis of the prognostic tests; Table S1: Results of statistical analysis (tumor-related mortality) of the 2011 two-tier histologic grading system; Table S2: Results of statistical analysis (all-cause mortality) of the 2011 two-tier histologic grading system.
