# Supplementary material for: Atypical Mitotic Figures Are Prognostically Meaningful for Canine Cutaneous Mast Cell Tumors
Source: Vet Sci. 2023 Dec 20;11(1):5. doi: 10.3390/vetsci11010005 (PMC10821277; doi:10.3390/vetsci11010005)
Supplement: Supplementary file 1 [file vetsci-11-00005-s001.zip › vetsci-2771361-supplementary.pdf]

**Supplemental material**

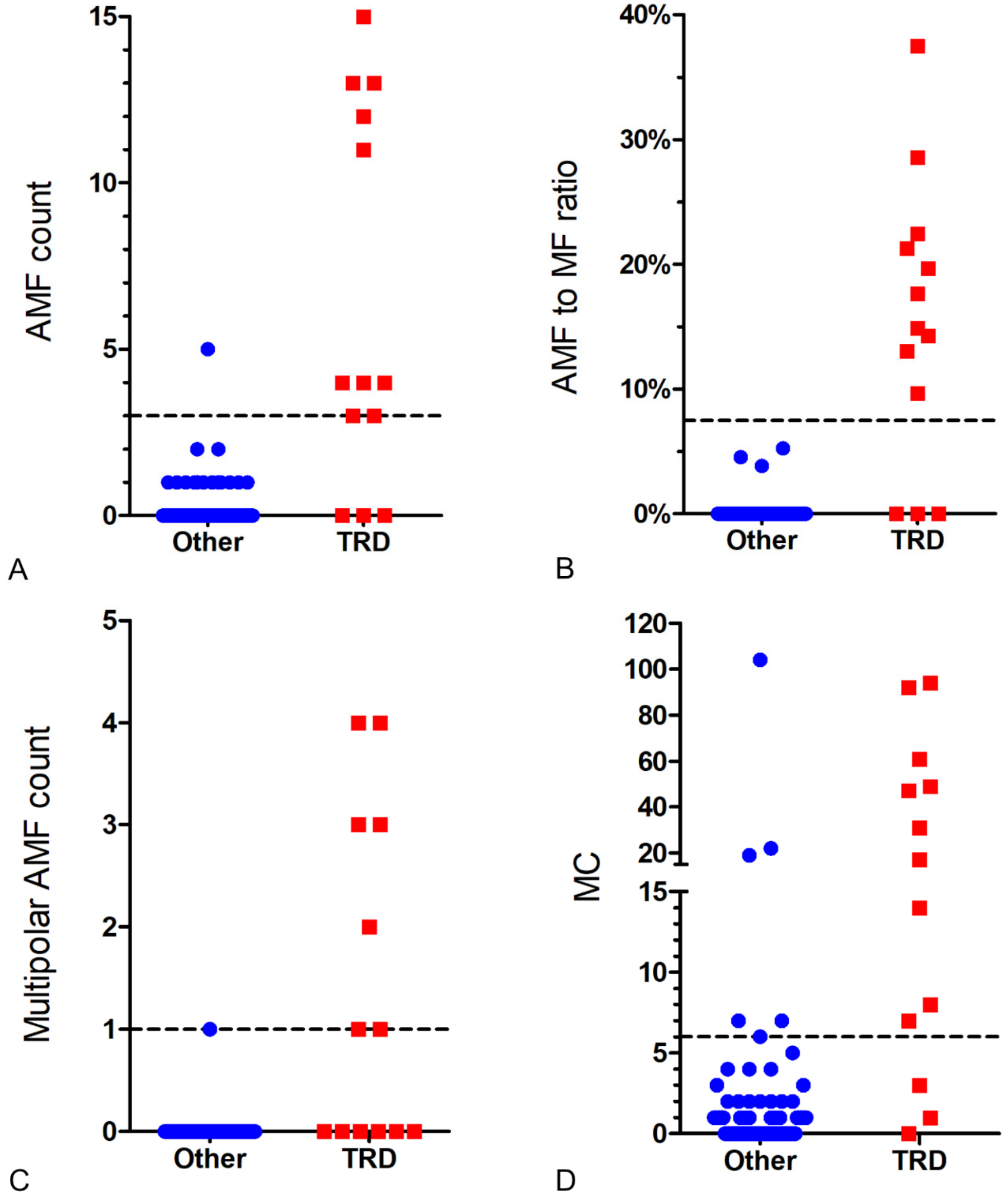


**Figure S1.** Scatterplot analysis of the prognostic tests comparing cases with tumor-related death (TRD) vs. other cases (without tumor-related death). A) Atypical mitotic figure (AMF) count. B) AMF to mitotic figure (MF) ratio. C) Multipolar AMF count. D) Mitotic Count (MC).

**Table S1.** Results of statistical analysis regarding the prognostic value (**tumor-related mortality / tumor-specific survival time**) of the 2011 two-tier histologic grading system.

| Statistical Analysis | Result |
| --- | --- |
| Sensitivity | 84.6% |
| Specificity | 91.6% |
| Positive predictive value | 61.1% |
| Hazard ratio (95% CI) | 46.5 (9.6 – 223.3) |

95%CI, 95% confidence interval

Only one canine cutaneous mast cell tumor had a mitotic count (MC) of 6, thus was predicted to have an aggressive biological behavior according to Romansik et al. and as low grade based on a MC threshold for the two-tier histological grading system (other grading parameters were low). This mast cell tumor was lost to follow-up at 18 months (i.e. correct prognostication based on the grading threshold). The three dogs with high grade mast cell tumors based on grading parameters other than the MC (i.e. karyomegaly and multinucleation) died due to the tumor after 2 months or died due to other cause 7 and 20 months after surgery. Thus, the sensitivity of the two-tier grading system (sensitivity: 84.6%) was higher as compared to the MC (sensitivity: 76.9%), whereas specificity was slightly lower (91.6% vs. 92.7%), due to the combination with other morphological criteria.

**Supplemental Table S2.** Results of statistical analysis regarding the prognostic value (**all-cause mortality at 12 months after surgery/ overall survival time**) of the 2011 two-tier histologic grading system.

| **Statistical Analysis** | **Result** |
|---|---|
| Sensitivity | 62.5% |
| Specificity | 90.0% |
| Positive predictive value | 55.6% |
| Hazard ratio (95% CI) | 11.1 (4.7 – 26.2) |

95%CI, 95% confidence interval
